# Book and Pamphlet Notices

**Published:** 1859-06

**Authors:** 


### BOOK AND PAMPHLET NOTICES.

A Practical Treatise on the Diseases of Infancy and Childhood. By T. H. Tanner, M. D., F. L. S., Licentiate of the Royal College of Physicians; late Physician to the Hospital for Women, etc., etc. Philadelphia: Lindsay & Blakiston, 1859.

This is a small work, but one of great value—treating upon a most interesting class of patients, and the diseases peculiar to them.

The importance of understanding, thoroughly, the diseases of infancy, cannot be easily over-estimated. The fact that in some of the public institutions of England, less than a century ago, *twenty-three* of every *twenty-four* infants, were, for a long time, allowed to die under one year of age, and another fact, that even now, in large cities, about one-fourth die before they attain their fifth year—demonstrate the importance of studying the diseases of infancy, and prove that much may be done by enlightened and well directed efforts to abate infant mortality, and also that much still remains to be done.

The practical teaching in this book is based, as it should be, upon the *peculiarities* of the organization of infants, a knowledge of which is the foundation of success in the treatment of this class of cases.

The chief structural differences are these: the tissues generally are softer, much more vascular, and more loaded with fluid; the glandular, lymphatic, and capillary systems predominate; the skin and mucous membranes are very delicate, soft, and sensitive; the brain is large and vascular, though so soft as to be almost fluid; and there is excessive nervous excitability. The term of infancy being essentially the period of growth, the organs of alimentation are those which are the most fully developed, as they are those which are the most actively employed; indeed, it may be said that at this age the functions are confined almost exclusively to nutrition.

Considerable space is devoted to the hygienic management of children, and the therapeutics of infancy, much of which we would gladly extract, had we room in the present number of the Journal, but must content ourselves with a few quotations from the treatment of some of the more important diseases. We will begin with scarlatina.

We have long doubted whether the the great fatality which so often attends scarlet fever, was an inevitable consequence of the nature of the disease; and have believed, that if the profession would regard as their duty, in self-limited diseases, simply to conduct to a favorable termination by preventing local complications, without attempting to abbreviate the disease by active interference, we might hope for a diminution in the fatality of this disease, as well as of the terror with which it is regarded by the public. We give the author's views of the treatment of scarlatina, as according very nearly with our own.

*Treatment.*—The treatment of scarlatina yet remains to be considered. The *simple form*, says Sydenham, is "fatal only through the officiousness of the doctor." It requires no treatment beyond confinement to the house for at least two or three weeks after all symptoms of the disease have disappeared, warm clothing, spare diet, and attention to the bowels. In *scarlatina anginosa* the treatment is often much the same as that for many cases of continued fever. Cold or tepid sponging where there is great heat; perfect ventilation of the sick-room, without draughts of cold air; emetics when the tongue is much coated, and nausea and irritability of the stomach exist; shaving the scalp, and the application of cold lotions where there is much delirium; great caution in the use of antiphlogistic medicines—as antimony; and a strict avoidance of bleeding, even by two or three leeches. Purgatives judiciously employed will often obviate the necessity for lowering measures of any other kind, but they must on no account be given too freely, or in too large doses. Saline medicines are grateful and cooling; or, where the pulse is feeble, effervescing draughts containing an excess of ammonia. When there are decided symptoms of depression or collapse, wine, cordial draughts of ammonia, æther, and camphor, and nourishing food, must be ordered.

In *malignant scarlet fever*, a stimulating plan of treatment, such as that so successfully pursued by Dr. Todd, myself, and others in typhus, alone offers any chance of success. The vital powers are so prostrated by the deadly force of the poison, that unless we support them by the free administration of brandy, wine, and bark, they will fail altogether. When seen early, however, the treatment may be advantageously commenced by a mild emetic. The gangrenous ulceration of the fauces, which often complicates this form, will be also best combated by the use of stimulants and the local application of a solution of the chloride of soda: but when very severe, the throat and fauces must be swabbed with a strong solution of nitrate of silver—gr. x. to ℥j. The chlorate of potash drink will be useful. Chlorine itself is used by some practitioners, who speak highly of its good effects, in even the worst cases. Belladonna, in very minute doses, has been recommended as a prophylactic against scarlatina. In an epidemic of this disease which occurred on board Her Majesty's ships "Agamemnon" and "Odin," in 1853, this remedy was freely tried, without the slightest benefit.

Upon the pathology and treatment of croup, we take the following:

*Pathology.*—From the foregoing it seems evident that this disease consists of inflammation of the mucous membrane, exciting spasmodic action in the larynx and trachea, and giving rise to a peculiar product—a psuedo-membranous secretion. The result is imperfect aeration of the blood, for the access of air to the minute vessels is impeded by the spasm as well as by the accumulation of the croupal productions.

The question as to whether the inflammation is of a simple kind, or dependent upon some specific poison in the system—as is the case with the eruptive fevers, etc.—has been entertained but not satisfactorily answered. My own views are certainly in favor of the latter opinion, and for these reasons:—the second attacks of croup are—as a rule—much less severe than the first, because the susceptibility of the system to the action of the poison is partly exhausted, just as is seen in the practice of syphilisation: the occasional prevalence of the disease in an epidemic form, seems to indicate a specific agent as its cause: when laryngitis is excited in children by some poisonous irritant, or by drinking boiling water from the spout of a tea-kettle, the results are very different, and false membranes are not exuded, even though the inflammation extend to the trachea.

When the inflammatory action is established, there are three remedies on which all authorities teach us to rely,—viz. blood-letting, tartar emetic, and mercury. Perhaps there is no infantile disorder which is so surely and early recognized by practitioners, and so zealously and perseveringly treated on this plan, as croup; for mistakes in diagnosis are very rare, and errors in treatment are seldom committed,—supposing that the authorities are correct. The question may well be asked then, —How is it that this disease is so fatal? I believe, from my own experience, because one of the chief agents is not only inappropriate but mischievous. Every physician knows that when he is summoned to a consultation on a case of croup, he is sure to find that the sufferer has been freely bled, either generally or locally; and he probably is informed that *in spite* of the loss of blood the inflammation increased. It never strikes the practitioner that he should say—"in consequence of;" yet it would probably be nearer the truth. I would strongly urge then, that this plan of indiscriminate bleeding be discontinued; that the case be fairly looked at, in all its bearings; that the sufferer's constitution and the condition of his vital powers be fully taken into consideration; and that we hesitate to deprive the flesh of that which we are told is its life, simply because there is inflammation of the air-tube, and books tell us to treat this dangerous affection by bleeding. Moreover we are taught to bleed very freely when the child is plethoric and robust, full of blood, full of life, healthy and vigorous, with all its powers active. But are such, the children who—generally speaking—suffer from this disease? Certainly not in town practice. And again, will bleeding render impure blood pure, or will it check inflammation? Is conjunctivitis controlled by the application of leeches round the orbit? does the vascularity decrease as the blood flows? I have never seen it do so. In rheumatic fever, which are the cases that are most liable to be affected with pericarditis or endocarditis? Is it not those that have been bled? Do women who have had natural labors suffer most frequently from puerperal metritis, or such as have had severe floodings? According to my own experience undoubtedly the latter. In short, I would counsel every practitioner to think and reason for himself: to scan every case of inflammation narrowly, and ask himself—Is there an excess of healthy blood here? and even if there be, will it not be required by-and-by to support the system under the prostrating effects of the disease? and finally—to consider well the result which has followed the employment of depletion when he has resorted to it or has actually witnessed its employment, and if he find that the good derived from the use of the lancet has been more than problematical, then I would advise him to throw the instrument away, let authorities say what they may.

But if we are not to bleed in a severe case of croup, what are we to do? When the patient is seen at the onset of the disease, the inflammatory action may sometimes be arrested by hot fomentations alone—as recommended by Dr. Lehman and successfully practised by Dr. Graves. A sponge, the size of a large fist, dipped in water as hot as the hand can bear, must be gently squeezed half dry, and instantly applied beneath the little sufferer's chin, over the larynx; the temperature being maintained by resoaking it every two or three minutes. A steady perseverance in this plan for twenty or thirty minutes, produces vivid redness of the skin over the whole surface of the throat; while under the influence of this topical treatment, a gentle perspiration breaks out—to be encouraged by warm diluents. A notable diminution also takes place in the cough, hoarseness, tone of voice, dyspnœa, restlessness, etc.; and generally a sound sleep is enjoyed, from which the patient awakes nearly well. Supposing that this amelioration does not take place, very little time has been lost, and we must resort to emetics—a most valuable class of remedies. The ipecacuanha wine—in doses varying from one drachm to two drachms, according to the age—should be given every fifteen minutes until free vomiting has been induced; and unless the breathing is relieved, a dose sufficient to keep up the nausea should be repeated every three or four hours until decided ease is afforded. When this is obtained, great benefit will result from the administration of a draught containing a little antimony, ipecacuanha, etc., every two or three hours; repeating the emetic of ipecacuanha every eight or ten hours according to the symptoms. In cases where prostration appears to be coming on, an emetic of alum or of sulphate of copper will be preferable to the ipecacuanha; while a mixture containing some ammonia and senega may also be judicially substituted for the antimonial medicine. At the same time that this plan is pursued, the temperature of the body is to be taken by a thermometer placed under the armpit, or with care under the tongue; and if—as it usually is in the first and second stages—the degree of heat is above the normal standard, a warm bath ought to be administered, and the patient immersed in it up to the chin for fifteen or thirty minutes, according to the effect produced. It is of course clear, that a patient having a temperature of 104° or 105° Fah., must part rapidly with some of this heat, if placed in water warmed only to 96° Fah.; unless as fast as the heat is given off it be regenerated. This bath is not only cooling, but sedative; it may be repeated twice or thrice in the twenty-four hours, but only under the personal superintendence of the practitioner. To avoid alarming the child, the bath should not be prepared in its presence; and when brought into the sick-room the top of it should be covered with a blanket, on which the patient can then be placed and slowly lowered into the water. A piece of wood or some toy may be floated on the surface, to engage the attention of the little sufferer.

Supposing that the disease advances notwithstanding these measures, or supposing that we only see the case when it has reached the end of the second stage, I resort at once to the use of the iodide of potassium in preference to any other remedy; and frequently—but especially if there be much depression—I combine with it some tincture of assafœtida and decoction of senega. I think also that the application of the compound tincture of iodine to the outside of the windpipe, about once in every twelve hours does good; and provided that it does not raise a blister, it can do no harm. If the practitioner have great faith in mercury preventing the formation of false membranes in croup, I would advise him not to omit the iodide of potassium, but to combine with it mercurial inunction, half a drachm or even a drachm being gently rubbed in every four or six hours.

No mention is made by the author of the remedy which we regard as unquestionably the most efficient in the treatment of croup. We refer to the hydrarg. sulph. flav. or turpeth mineral. This medicine is so safe and certain in its operation in this disease, that we have been in the habit of using it for a number of years, and rely almost exclusively upon it; and from experience with it, we commence the treatment of a case of croup with nearly the same confidence that we would an intermittent. It may be given in any stage of the disease, agreeably to the directions of the U. S. D.

From the severity of the symptoms sometimes attending ulceration stomatitis, and from the ease with which it is managed, we extract the following:

*Ulcerative Stomatitis, or Noma.*—This disease attacks the gums; the ulceration sometimes progressing to such an extent as to destroy these parts and denude the teeth. In all cases it produces heat of the mouth, an increased flow of saliva, an offensive breath, swelling of the upper lip, and enlargement with tenderness of the submaxillary glands: while on looking in the mouth we shall see that the gums are swollen, red or violet colored, readily bleeding to the touch, and covered with a layer of pulpy greyish matter. If the disease be allowed to creep on unchecked, the gums will be destroyed by the ulceration, and the teeth become exposed and loosened until they fall out; the morbid action also spreads to the inside of the cheeks, which become covered with irregular sloughing ulcerations, and the tongue assumes a swollen and sodden appearance. Ulcerative stomatitis is not uncommon: it occurs for the most part in weakly children who have been badly nourished, and exposed to cold and damp.

The *treatment* of this disease is not difficult, inasmuch as we possess in the chlorate of potash a remedy which may almost be deemed a specific. Five grains of this salt may be given every four or six hours to an infant one year old, in a little sugar and water. When the ulcerations have healed, bark or quinine should be administered.

This book would be a valuable addition to the library of any physician. For sale by W. B. Keen, 148 Lake street. M.

---

First Annual Report of the Chicago Charitable Eye and Ear Infirmary. Chicago, 1859. James Barnet, Printer.

OFFICERS.

WALTER L. NEWBERRY, President.

CHARLES V. DYER, } Vice-Presidents.

LUTHER HAVEN, }

SAMUEL STONE, Secretary.

EDWARD L. HOLMES, Treasurer.

TRUSTEES.

| | |
|---|---|
| WALTER L. NEWBERRY, | FLAVEL MOSELEY, |
| WILLIAM H. BROWN, | SAMUEL STONE, |
| CHARLES V. DYER, | PHILO CARPENTER, |
| LUTHER HAVEN, | REV. N. L. RICE, D.D., |
| EZRA B. McCAGG, | JOHN H. KINZIE, |
| WILLIAM BARRY, | MARK SKINNER. |

BOARD OF SURGEONS.

| | | |
|---|---|---|
| *Consulting Surgeons.* | PROF. DANIEL BRAINARD, M. D., | *Trustees Ex Officio.* |
| | PROF. JOSEPH W. FREER, M. D., | |
| *Attending Surgeons.* | EDWARD L. HOLMES, M. D., | |
| | WILLIAM H. BALTZELL, M. D., | |

The number of cases treated during the past year, which is the first, is 115—of which, 95 were of the eye, and 20 of the ear. The following extracts from the report will exhibit the objects and necessity of its being established:

Every philanthropist must rejoice, that the blind are furnished by the public bounty with such an institution. Its establishment reflects honor upon the State, and it should be maintained at any cost. But how much more in accordance with an enlightened humanity, and, in an economical view, how much more reasonable and advantageous, to provide the necessary means, comparatively so small, for institutions devoted to the *prevention* of blindness—and thus, in the most effective manner, avert the misery which must always attend it, as well as the burdens, private or public, it must entail.

In conclusion, the surgeons would again particularly impress upon the minds of the trustees and the public, that charities of this kind are not designed for the support of those who have become helpless, and hopelessly dependent, from the effects of vice and disease, and who are always maintained at a large expense. They have for their object, the prevention of one of the greatest calamities which can befall a human being—blindness; the sad consequences of which, to their full extent, it is hardly in the power of the imagination to conceive, still less, to portray.

We only deem it useful to say in behalf of this charity, that it is under the management of Drs. Holmes and Baltzell, who are entirely competent to take charge of it, and we recommend physicians who may have occasion to give advice to those laboring under diseases of the eye and ear, in regard to those to whom they should apply, to direct them to these gentlemen. They will thus not only do them a service, but be the means of keeping them out of the hands of those who circulate advertisements to entrap the unwary, and who are but too ready to take advantage of the hopes and credulity of the unfortunate.

